# Bacterial Toxins for Cancer Therapy

**DOI:** 10.3390/toxins9080236

**Published:** 2017-07-28

**Authors:** Nour-Imene Zahaf, Gudula Schmidt

**Affiliations:** Institute for Experimental and Clinical Pharmacology and Toxicology, Faculty of Medicine, Albert-Ludwigs-University, Albert-Str. 25, 79104 Freiburg, Germany; nour.imene.zahaf@pharmakol.uni-freiburg.de

**Keywords:** bacterial toxin, cancer, specific transport, immunotoxin, targeted toxin

## Abstract

Several pathogenic bacteria secrete toxins to inhibit the immune system of the infected organism. Frequently, they catalyze a covalent modification of specific proteins. Thereby, they block production and/or secretion of antibodies or cytokines. Moreover, they disable migration of macrophages and disturb the barrier function of epithelia. In most cases, these toxins are extremely effective enzymes with high specificity towards their cellular substrates, which are often central signaling molecules. Moreover, they encompass the capacity to enter mammalian cells and to modify their substrates in the cytosol. A few molecules, at least of some toxins, are sufficient to change the cellular morphology and function of a cell or even kill a cell. Since many of those toxins are well studied concerning molecular mechanisms, cellular receptors, uptake routes, and structures, they are now widely used to analyze or to influence specific signaling pathways of mammalian cells. Here, we review the development of immunotoxins and targeted toxins for the treatment of a disease that is still hard to treat: cancer.

## 1. Introduction

Immunotoxins (IT) are chimeric proteins developed for specific targeting of cancer cells. These molecules are composed of two major parts: a receptor-binding moiety, which is in most cases an antibody (from which the name immuno-toxin is derived) or a ligand (targeted toxin) directed towards a specific receptor expressed on the cell membrane. The second part of the IT is usually the catalytic part of a toxin, an enzyme, responsible for the toxin-induced lethality [1]. 

Monoclonal antibodies are commonly used for cancer treatment. They have proven to be efficient with a prolonged overall survival among breast cancer patients [2], or among people suffering from leukemia [3]. However, especially for solid tumors, still continuous improvement of treatment is required. Immunotoxins use the specificity of the antibodies produced towards target molecules on the cell membrane. This presents an opportunity to deliver toxic components into the cell.

The antibody part of the IT is commonly shortened to its antigen-binding domain, in order to decrease immunogenicity [4]. The catalytic part is genetically engineered to improve toxic activity and/or to decrease antigenicity [4]. When bound to the targeted receptor, the immunotoxin is endocytosed. To modify its molecular target, the toxin has to be released into the cytosol. Many toxins have the intrinsic capacity to escape to the cytosol following acidification of the endosomes and pore formation. Others take the route back through the Golgi body and endoplasmic reticulum into the cytosol [5,6,7]. Within the cytosol, the catalytic part of the toxin modifies its target, ultimately triggering cell death. The most commonly used toxins for the generation of immunotoxins are of plant origin, for example, gelonin and ricin [8], or from bacteria, such as diphtheria toxin and *Pseudomonas* exotoxin A. All these toxins have been extensively studied and are well characterized. The two bacterial toxins showed to be extremely efficient in killing cells. They both irreversibly modify the mammalian elongation factor 2 (eEF2). Thereby, they inhibit protein translation and induce cell death [9].

The focus of this review is on bacterial toxins used for the generation of immunotoxins and targeted toxins. 

## 2. Single Chain Bacterial Toxins

The idea behind targeted toxins is to combine the specificity of a ligand towards a surface receptor highly expressed on cancer cells with the toxins’ catalytic part for killing tumor cells. One of the first bacterial toxins used for this purpose was diphtheria toxin, the primary virulence factor produced by *Corynebacterium diphtheria*. Diphtheria toxin has been extensively studied and excellent reviews about the mechanism of action of this toxin are available [10,11]. The crystal structure of the diphtheria toxin uncovers a single chain, a Y-shaped protein formed by two distinct parts [12]. The A part is an enzyme with an ADP-ribosylating activity. It catalyzes the ADP-ribosylation of the mammalian elongation factor eEF2, leading to its inactivation and blockage of the aa-tRNA in the A site of the ribosome. The toxin needs nicotinamide adenine dinucleotide (NAD^+^) as a second substrate [13]. Toxin-catalyzed ADP-ribosylation occurs at diphthamide, which is a modified histidine amino acid of eEF2. ADP-ribosylation of eEF2 blocks protein synthesis and causes cell death [14]. Part B (C-terminal) is formed by two domains: a transmembrane domain built of nine α-helices, which triggers a pH-dependent membrane insertion; and a receptor-binding domain. The receptor-binding domain interacts with the heparin-binding EGF-like growth factor (HB-EGF) on the surface of targeted cells and assists the passage of fragment A (via the translocation domain) into the cytosol [12]. Diphtheria toxin is extremely toxic. Only one single molecule reaching the cytosol is required to kill a cell [15]. 

**Figure 1:**
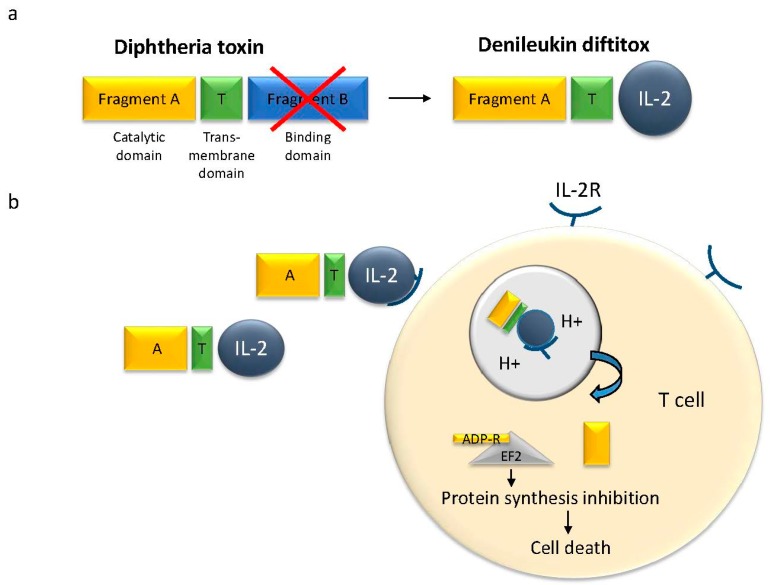
Denileukin diftitox (structure and action). (**a**) The diphtheria toxin is formed by three domains: a catalytic domain (A), a trans-membrane domain, and receptor-binding domain. In Denileukin diftitox the receptor binding domain has been exchanged for human interleukin 2 (IL-2) for a specific interaction with the IL-2 receptor; (**b**) Denileukin diftitox recognizes the IL-2 receptor on the transformed T-cells and is internalized to the endosome. After acidification of the endosome, the catalytic part is released into the cytosol and ADP-ribosylates the elongation factor 2 (EF-2), thereby inhibiting protein synthesis and causing cell death.

Because of this high toxicity, diphtheria toxin was selected for the generation of the first immunotoxin, called *Denileukin Diftitox* (Ontak, Figure 1). It is also the first immunotoxin approved by the Food and Drug Administration (FDA) for the treatment of cutaneous T-cell lymphoma (CTCL) [16]. It was specifically redirected towards cancer cells by linkage of the fragment A to recombinant human interleukin-2 (IL-2), in order to target the IL-2 receptor, which is highly expressed on malignant T-cells [17,18]. Ontak showed good efficiency against CTCL and surprisingly, no antibodies against Ontak formed [19]. However, use of the targeted toxin produced severe side effects, because the receptor is not exclusively expressed on the malignant T-cells. The most common side effects related to *Denileukin Diftitox* treatment are blurred vision or disruption of color vision, nausea, diarrhea, skin rash, and muscle pain. Some other effects are flu-like symptoms and, most importantly, vascular leak syndrome (VLS) [20]. In 2006, the FDA added a black box warning label to Ontak. Bacterial expression systems generate only low amounts of the IL-2 fusion toxin. In a recent publication, Wang et al. reported on a change of the expression system to yeast [21]. The purified “yeast-Ontak” showed high efficacy in a mouse model.

A second single chain toxin, which also blocks protein synthesis in mammalian cells, is Pseudomonas aeruginosa exotoxin A (PE) [22]. Like diphtheria toxin, it is highly toxic for mammalian cells. PE is a typical AB toxin formed by two domains. The A domain is responsible for the catalytic activity, while the B domain is a receptor binding moiety, which interacts with the low-density lipoprotein receptor-related protein 1 (LRP1 or CD91) [23]. LRP1 is also the receptor of Clostridium perfringens toxin TpeL [24]. Like the diphtheria toxin, PE is an ADP-ribosyltransferase which catalyzes the modification of elongation factor eEF2 in diphthamide to block protein synthesis and thereby induces cell death [25]. The domain structure of PE offers many possibilities for genetic engineering. Indeed, several immunotoxins have been generated by the exchange of the receptor binding part of PE to antibodies or ligands, which have been shown to interact with the specific receptors on the cell surface [26,27]. PE needs activation by proteolytic cleavage catalyzed by furins. Furins are subtilisin-like cell surface serine proteases involved in the processing of many cellular proteins. They are widely distributed in the human body. Diverse bacterial toxins use furins for their direct activation on the cell surface. Extensive analysis of PE by Pastans’ group identified a 38 kDa fragment of the PE toxin which does not require a furin cleavage and which possesses an increased toxicity to cancer cells [28]. Since, PE38 is used frequently for the generation of immunotoxins (for review see [29]). The primary aim of constructing different PE38 based immunotoxins was to target different tumor entities by exchange of fused antibodies or receptor ligands. The second aim was to reduce immunogenicity because the generation of anti-drug antibodies neutralized the activity of the immunotoxin and limited repeated treatments for a cancer patient.

Meanwhile, several immunotoxins based on PE38 are in preclinical or clinical evaluation. For example, PE38 has been fused to a single chain antibody, which specifically targets the glycoprotein non-metastatic gene B (NMB) for the treatment of malignant gliomas and melanomas [29]. A disulfide-stabilized immunotoxin was generated for targeting osteosarcoma using an antibody that targets the antigen associated with osteosarcoma (TP-3) [30]. The immunotoxin BL22 has completed a phase I clinical trial. An anti-CD22 single-chain antibody fused to PE38 forms this toxin. The protein targets CD22, which is overexpressed in B cell tumors. BL22 was the first drug, which resulted in a complete remission of patients with purine analog resistant hairy cell leukemia (HCL) [31].

BL22 was later replaced by a second-generation *Pseudomonas* exotoxin A-based immunotoxin targeting CD22: HA22. This toxin is a mutant of BL22 with mutations in the heavy-chain domain 3 resulting in an increased cytotoxic activity. In order to improve the half-life of the BL22 immunotoxin, Bang and coworkers additionally mutated Arg 490 to Ala, because this residue is involved in proteolytic digestion and, therefore, results in rapid degradation of HA22 [32]. The mutant showed resistance to proteolytic degradation and improved efficacy; it was highly effective in killing B cells from a variety of tumor cell lines. Viability assays showed that the stabilized HA22 is 10 times more potent than BL22 [32].

The genetic engineering of immunotoxins to decrease immunogenicity has been a major research topic and has proven to be quite successful. Recently, T-cell epitopes have been mutated, producing next generation immunotoxins with lower immunogenicity [33]. Moreover, PEGylation severely changes pharmacokinetic properties and immunogenicity of proteins or peptides and has been used to reduce immunogenicity of PE-based immune-conjugates. However, this modification reduced the activity of the toxin [34]. Therefore, a lot needs to be done to completely overcome the development of anti-drug antibodies. 

## 3. Pore Forming Toxins

**Figure 2:**
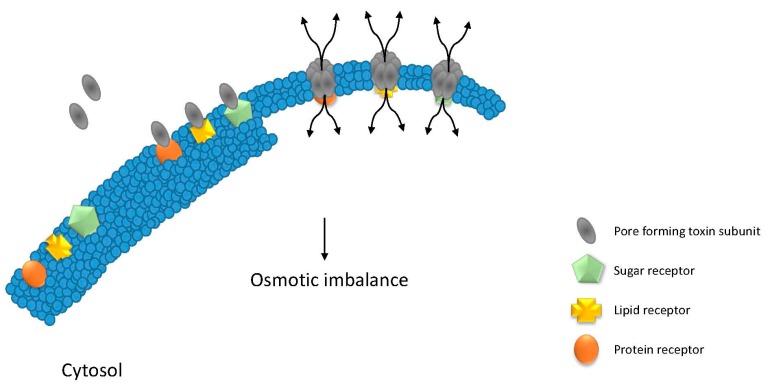
Pore forming immunotoxin mode of action. The pore forming toxins oligomerize on the cell surface after recognition of their receptors, which are sugars, lipids or proteins, respectively. When the pore is formed, it inserts into the plasma membrane allowing the free passage of electrolytes and other small molecules, creating an osmotic imbalance.

Toxins differ in their structure, activity, and mode of action. Some toxins are different from single chain toxins. They do not need a catalytic domain for toxicity. These toxins are called pore-forming toxins (PFTs, Figure 2). PFTs insert into the plasma membrane to short-circuit the membrane potential. They bind to their target receptors, which can be sugars, proteins, or lipids. After binding, the PFTs oligomerize to form a pore inserting directly into the plasma membrane [35]. Many efforts have been made to develop immunotoxins based on pore forming toxins (pore forming immunotoxins (PFITs)). One of the first developed pore-forming immunotoxins was generated by fusion of cytolysin equinatoxin II—isolated from the sea anemone *Actinia equina* L—to human transferrin, which is a regulator of cellular growth [36,37]. Transferrin was an interesting ligand candidate because its receptor was thought to be only present on transformed, activated, and malignant cells [38].

Although this immunotoxin was efficient against tumor cells in vitro, it maintained an unspecific hemolytic activity due to the fact that it preserved its ability to unspecifically bind to cell membranes [39]. Many pore-forming immunotoxins have been developed in this manner [40]. The fact that pore forming toxins interact with cell membranes remains, however, an obstacle to their usage. Alpha-hemolysin, the major toxin produced by *Staphylococcus aureus*, shows less unspecific membrane interaction. It consists of a single-chain, hydrophilic molecule which has to be activated by proteolytic cleavage before insertion into the membrane. This cleavage is physiologically catalyzed by A-disintegrin and metalloprotease 10 (ADAM10), present on many mammalian cells [41]. Specificity against tumor cells was enhanced by mutagenesis of the protease recognition site towards structures cleaved by matrix metalloproteases (MMPs) which are secreted by tumor cells in large amounts to destroy the extracellular matrix [42,43]. The hexamers of the alpha toxin generate non-selective pores 1–2 nm in diameter that permit free passage of ions and low-molecular-mass molecules. The N- and C-terminal domains of the α-toxin are connected by a central glycine-rich loop [44]. 

Although several efforts have been made for the design of pore-forming immunotoxins, their usage is still not obvious. However, the PFITs, may be used for combination therapy with chemotherapeutic drugs since their ability to form pores facilitates the entry of other drugs into cancer cells. 

## 4. Anthrax Toxin as a Transporter

Re-routing toxins towards specific receptors of target cells has proven to be successful. The uptake of the immunotoxin into the cytosol of the targeted cell has been a major obstacle [45,46,47,48]. Indeed, most of the toxins need translocation through the endosomal membrane to reach their substrate in the cytosol [49]. The catalytic part of the toxin linked to an antibody may be not sufficient to translocate it through the endosomal membrane. Single chain bacterial toxins encompass an additional translocation domain, mediating membrane transition. Therefore, toxins, which are composed of two separate proteins, were used to generate a novel kind of targeted toxins. One protein is the cell binding and pore-forming moiety, which is redirected towards cancer cells. The second protein is catalytically active and recruited by the pore-forming part. The pore is formed in the endosomal membrane following acidification of the endosome and is used to translocate the enzyme into the cytosol. A member of this group of two chain toxins is the anthrax toxin. Anthrax toxin is an AB type toxin secreted by *Bacillus anthracis*, a Gram-positive bacterium responsible for the anthrax disease. It consists of three non-linked compounds, which are separately non-toxic [50]. The protective antigen (PA) is the cell binding and pore forming part of the toxin. It is an 83 kDa protein which binds to the tumor endothelial marker 8 (TEM8) or the capillary morphogenesis gene 2 (CMG2), called anthrax receptors 1 and 2, respectively [50]. The crystal structure of PA has been solved [51]. It is formed by four domains; containing the furin cleavage site for activation and the endosomal membrane insertion site which is important for the release of effectors into the cytosol. The third domain is responsible for the oligomerization of the PA monomer after cleavage, and the fourth domain is the receptor-binding domain.

The fourth domain has to be modified to generate specificity towards diverse tumor cells. Cleavage of the PA by furins or furin-like proteases enables binding to its receptors followed by oligomerization. Following cleavage, a 20 kDa protein is released and the remaining 63 kDa protein oligomerizes to form a pre-pore, unveiling the binding site from the lethal factor (LF), a 90 kDa protein, and the edema factor (EF), an 89 kDa protein.

The internalization of the Anthrax toxin complex is mediated by clathrin-dependent endocytosis [52]. Following acidification of the endosome, the PA pre-pore is inserted into the endosomal membrane to form a pore. Bound LF and EF unfold and are delivered into the cytosol where they refold and modify their respective targets. The lethal factor is a metalloprotease, which degrades MAPK kinases [53,54], and EF is a calmodulin-dependent adenylyl cyclase, which catalyzes the production of cAMP, responsible for the generation of edema [55].

PA is a key component of the anthrax toxin, mediating cellular contact. Similar to the work on PE, two different properties of protective antigen have been modified to reach higher specificity towards cancer cells:

First, as described for the pore-forming toxins, the proteolytic activation of PA by furins for activation has been exploited. The furin cleavage site of the PA was changed to a matrix metalloprotease (MMP) cleavage site because MMPs are frequently overexpressed by cancer cells [56]. These MMP-targeted PA proteins were selectively activated by MMP-overexpressing tumor cells, while the toxicity on cells not expressing MMPs was at least 100-fold lower.

Second, the receptor binding properties of PA were successfully changed. Following extensive analysis of the toxin-receptor interaction and successful crystallization of the toxin oligomer, the specificity of PA was changed by creating two mutations in the receptor binding domain of PA (N682A and D683A), avoiding the binding to its native receptors. Subsequently, the binding properties of the mutant PA were changed by fusing a cellular receptor ligand. For example, epidermal growth factor (EGF) was linked to the mutated PA in order to redirect the PA towards the epidermal growth factor receptor (mPA-EGF) [57]. The EGFR is frequently overexpressed on many cancer cells [58]. The same group followed up with a new transporter redirected against the human epidermal growth factor receptor 2 (HER2), which is often overexpressed in breast and ovarian cancers [59]. The new transporter consists of the mutated PA (N682A/D683A) fused to ZHER2, an affibody (small protein binding a target molecule with high affinity and specificity), that binds with high affinity to HER2 (mPA-ZHER2).

**Figure 3:**
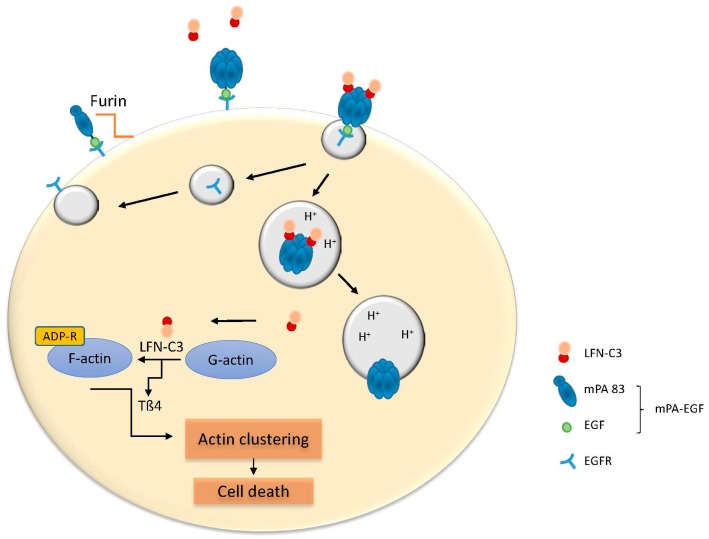
Modified anthrax protective antigen (PA)-mediated endocytosis of LFN-C3 (anthrax lethal factor-N-terminus fused to the catalytic domain of *Photorhabdus luminescence* TccC3 toxin). The mPA-EGF monomer recognizes the EGF receptor on the cell surface where it forms a pre-pore following activation by furin-like proteases. The LFN-C3 part binds to the pre-pore and the complex is endocytosed. After acidification of the endosome, LFN-C3 is released into the cytosol and ADP-ribosylates actin, thereby disrupting the cell cytoskeleton.

Two-component toxin allows not only the redirection of the toxin transporter, but also the exchange of the catalytic part. LF and EF bind to the anthrax pore with the N-terminus, which alone has no catalytic activity and is sufficient to direct diverse proteins through the pore. Therefore, the fusion of the N-terminal peptide to other toxins or toxin fragments enables their delivery into mammalian cells through the anthrax PA. For example, the N-terminal part of the anthrax lethal factor (LFN) was linked to the catalytic part of the diphtheria toxin (LFN-DTA). Both redirected toxin transporters (mentioned above) enable the delivery of the toxic diphtheria component with high specificity into EGFR or HER2 overexpressing cells and induce death of the tumor cells [60]. More recently, the transport of a new chimeric effector via the mPA-EGF and mPA-ZHER2 transporters was shown (Figure 3). This new chimera consists of the N-terminal part of LF linked to the catalytic part of an effector from the *Photorhabdus luminescence* toxin TccC3hvr (aa 679–960) [61]. TccC3 is an ADP-ribosyltransferase, which modifies actin at threonine 148. This modification blocks the interaction of actin monomers with thymosin-β4, causing uncontrolled acting clustering and cell death. The LFN-C3 construct was transported selectively into the EGFR-overexpressing mid-esophageal cells OE21 via the mPA-EGF transporter and in the HER2 overexpressing low esophageal OE33 cells via the mPA-ZHER2 transporter, selectively eliminating the targeted cells [61]. Mouse studies are definitely required to validate the specific action of these targeted toxins in vivo.

## 5. Testing Immunotoxins In Vivo

ITs were tested in vivo for toxic effects and immunogenicity. In early studies, the action of an immunotoxin composed of *Pseudomonas* toxin combined with an antibody against the human transferrin receptor was analyzed. Mice were injected with ovarian cancer cells followed by the immunotoxin after five to eight days. Mice injected with the immunotoxin survived one month longer than control mice, which died in one to two months following injection of tumor cells [62]. This study showed that ITs could be used as an effective tool for targeting specific cancers. Targeting the human transferrin receptor was secondary in this study. Meanwhile, the receptor has been found to be expressed in several normal tissues other than cancer cells. However, the study suggested low toxicity of the immunotoxin in mice. 

In a study by Bumol et al. Diphtheria toxin fragment A was conjugated to a monoclonal antibody against chondroitin sulfate proteoglycan of human melanoma. The immunotoxin was compared to the monoclonal antibody alone. Results showed that only the DTA immunotoxin conjugate could inhibit in vitro protein synthesis and M21 cell growth, whereas both were potent at inhibiting growth in vivo [63]. The resulting effect of the monoclonal antibody in vivo was thought to be mediated by specific antibody-dependent cellular toxicity. This study shows the importance of in vivo testing since several factors could affect the potency and toxicity of ITs in vivo. However, studies in mice are limited, because the binding partner of ITs are often of human origin. Therefore, adverse effects in mice do not necessarily represent adverse effects in humans. Denileukin diftitox was tested for its ability to suppress regulatory T-cells (Tregs). Tregs are associated with poor prognosis due to a decreased antitumor response in patients. Tregs suppress the effect of proliferative T lymphocytes [64]. In mice, a single injection with the immunotoxin resulted in long-term depletion of Tregs [65]. These results are, however, contradictory with a clinical study published previously: neither an effect on the reduction of Tregs nor regression of metastatic melanoma was observed after Denileukin diftitox treatment of human patients [66]. A study by Yamada et al. showed that Denileukin diftitox induces a short-term reduction of regulatory T cells in non-human primates. However, they detected a severe toxic effect on natural killer (NK) cells, which may explain the weak effect of the agent in patients [67]. This, once more, shows the importance of testing immunotoxins in vivo.

## 6. Future Perspectives

Nature provides us with many different bacterial toxins with high efficiency and specificity towards central cellular molecules. Therefore, these toxins are frequently used to study specific signaling pathways or to manipulate the behavior of mammalian cells. By fusion to specific antibodies directed against tumor cells, immunotoxins have been redirected to cancer cells. A similar idea was the basis for the development of targeted toxins. In these artificial molecules, the binding site of the toxins to the natural receptor is replaced by a ligand, which interacts with a cell surface receptor strongly expressed on tumor cells. 

One of the limitations for the successful use of immunotoxins and targeted toxins is the fact that they are foreign proteins, which induce the generation of neutralizing antibodies in the patient. However, a deep understanding of uptake mechanisms, receptor binding, and molecular structures has allowed us to generate smaller, less immunogenic immunotoxins—for example, by removing T cell epitopes—with higher chances for re-treatment of patients. 

A second limitation is the specificity towards cancer cells. The efficiency is often so high that healthy cells may also be harmed. Therefore, less toxic molecules—e.g., toxins which block the movement of cells (less toxic for healthy cells)—may be used to interfere with metastasis, which is the most destructive process of cancer development.

A third challenge is the treatment of solid tumors. They are a pool of cells with different properties, including cells with different surface molecules. Therefore, the immunotoxins are not delivered to all cells of the tumor and complete clearance of all cancer cells is unlikely. Recent reports showed bacterial infiltration of solid tumors in colon cancer. Those tumors may be targeted by engineered toxin-producing bacteria, which locally produce toxins or which need a direct contact to the tumor cells to deliver the toxic moiety by a needle-like apparatus, the type 3 secretion system [68].

Altogether, the idea of using bacterial toxins for tumor therapy is still attractive but it needs further creative work for better success.
